# The role of the cortex in indentation experiments of animal cells

**DOI:** 10.1007/s10237-022-01639-5

**Published:** 2022-10-25

**Authors:** Leszek Krzemien, Magdalena Giergiel, Agnieszka Kurek, Jakub Barbasz

**Affiliations:** 1 Jerzy Haber Institute of Catalysis and Surface Chemistry, Polish Academy of Sciences, Niezapominajek 8, 30239 Krakow, Poland; 2 Department of Physics of Nanostructures and Nanotechnology, Institute of Physics, Jagiellonian University, Prof. Stanislawa Lojasiewicza 11, 30348 Krakow, Poland

**Keywords:** Cell structure, Finite element method, Elasticity, Atomic force microscopy

## Abstract

We present a model useful for interpretation of indentation experiments on animal cells. We use finite element modeling for a thorough representation of the complex structure of an animal cell. In our model, the crucial constituent is the cell cortex—a rigid layer of cytoplasmic proteins present on the inner side of the cell membrane. It plays a vital role in the mechanical interactions between cells. The cell cortex is modeled by a three-dimensional solid to reflect its bending stiffness. This approach allows us to interpret the results of the indentation measurements and extract the mechanical properties of the individual elements of the cell structure. During the simulations, we scan a broad range of parameters such as cortex thickness and Young’s modulus, cytoplasm Young’s modulus, and indenter radius, which define cell properties and experimental conditions. Finally, we propose a simple closed-form formula that approximates the simulated results with satisfactory accuracy. Our formula is as easy to use as Hertz's function to extract cell properties from the measurement, yet it considers the cell’s inner structure, including cell cortex, cytoplasm, and nucleus.

**Supplementary Information:**

The online version contains supplementary material available at 10.1007/s10237-022-01639-5.

## Introduction

Despite the improvements in the available equipment, measuring the mechanical properties of animal cells is challenging. There are several measurement techniques precise enough to deform a single cell in a controlled way and read out the force used in the process. They include atomic force spectroscopy, optical traps, micropipette aspiration, among other techniques. (Lim et al. 2006a, b). Such experiments determine the apparent Young’s modulus. That means that the results of different experiments cannot be properly compared. They depend on the type of experiment and even the geometrical properties of the equipment, e.g., the shape of the indenter in case of the AFM indentation (Calabri et al. 2006; Chen 2014). To extract the mechanical properties of the cell, a model is needed to describe the details of probe-cell interactions. This is not a trivial task because a cell is a complex structure consisting of multiple distinct parts.

The component responsible for most of the mechanical interaction between the cell and the environment is the cytoskeleton. It is a scaffold-like structure of protein filaments bathed in the cytoplasm. It is not practical to model the cytoskeleton in its whole complexity. Instead, some simplifications have to be made. It appears that for spherical cells it is best to divide the cytoplasm region into two parts: the dense, stiff outer layer of the cortical cytoplasm and the softer inner layer. The cortex has typically a thickness of several hundred nanometers, which is not insignificant compared to the other components of the cell. Therefore, it has to be modeled as a three-dimensional body to account for its nonzero bending stiffness. Such thin 3D structures, called shells, are nicknamed the *prima donnas* of structures in the engineering community (Ramm and Wall 2004), as there are no mathematical methods that would allow the derivation of an analytical formula properly describing their behavior. Finite element modeling is often the method of choice for thin 3D objects.

In this work, we present a finite element model of an animal cell, with the cortex treated as a 3D material, subjected to the AFM measurement with a spherical indenter. The analysis of the results allows for the development of a simple, closed-form formula that approximates the simulation in a reasonable range of parameters with satisfactory accuracy. Such a formula can be very useful in extracting material properties from AFM measurements.

Section 2 contains the research background, review of the literature, and the most essential experimental and theoretical results. Section 3. Presents the general assumptions of our model, the methods used, and crucial observations. Our calculations are presented and discussed in Sect. 4. Conclusions and closing remarks are given in Sect. 5.

## Theory and Calculations

### Multilayer cell elasticity models

In our approximation, a cell is composed of three key structures that may affect its mechanical properties. These elements are:

- Cortex—the membrane skeleton, located on the inner face of the cell membrane composed mostly of actin filaments;
- Cytoplasm—the substance filling the interior of the cell, surrounding the organelles and the cytoskeleton, which is a rigid structural lattice made of microfilaments, intermediate filaments, and microtubules;
- Nucleus—composed of nuclear envelope, nuclear lamina, nucleolus, chromosomes, nucleoplasm, etc.

The cell membrane, the vital element of each cell (made up of an extremely thin layer of phospholipids and cholesterol), is characterized by relatively low stiffness. Therefore, it was omitted due to a negligibly small contribution to the overall elastic response. To ensure this approach was correct, we examined the influence of a 10 nm thick bilipid layer on cell stiffness. The results confirmed that neglecting the cell membrane did not alter the simulation noticeably.

Modeling a cell requires a fine balance between accuracy and performance. The more elaborate the model, the more detailed predictions it can give, at the expense of computation time. Moreover, more complex models are more difficult to use, since they require more knowledge from users about the details of an object**.** Bursa et al. (2006) in their review mention that so far three main types of modeling have been used:

- Continuum nonstructural = cell is a homogenous, isotropic material;
- Continuum structural = cell has a structure, but each component is homogenous;
- Discrete structural = cell is represented as a system of discrete elements (for example, a cytoskeleton model).

All these approaches have been exploited in the literature. Bursa and Fuis (2010) presented a three-dimensional model of eukaryotic cells consisting of the cell membrane, cytoplasm, cytoskeleton, and nucleus. Due to the complex structure, load transmission can be simulated with more accuracy. We take a similar approach, constructing a continuum structural model.

The experiments suggest that the elasticity of cells depends on their shape (Melzak and Toca-Herrera 2015). Caille et al. (2002) model a cell as a body consisting of two components—cortex and nucleus. They show differences between round and spread cells. For the cells discussed in that paper, the estimated parameter of elasticity of round cells is 323 Pa, while spread cells have Young’s modulus of 775 Pa. There is also a distinction between the estimated values of the elasticity parameter for the nucleus inside the cell (about 5 kPa) and the isolated nucleus (about 8 kPa).

The structure of the cytoskeleton influences mechanical properties and mechanotransduction processes. The impact of the cytoskeleton stiffness was presented by comparing normal osteoblast cells and osteosarcoma cells (Wang et al. 2016). Young’s modulus of normal osteoblasts is greater than osteosarcoma-2.42 (1.08) kPa versus 0.82(0.43) kPa, which confirms that cancer cells have a reduced filament structure of actin. A decrease in stiffness is also observed after cells are treated with cytochalasin D, which disrupts the assembly of the F-actin filament.

The comparison between finite element analysis and experimental results was conducted for chondrocytes (cells in articular cartilage) using the method of micropipette aspiration (Baaijens et al. 2005). This study was conducted to determine whether the key factor of the behavior is attributed to the intrinsic viscoelasticity of the cytoplasm or the biphasic effect of fluid–solid interactions between the cells. The results conclude that intrinsic viscoelastic phenomena are more important than biphasic behavior.

A comprehensive review of the cell models was given in the paper by Lim et al. (2006a, b). The authors of the review point out the lack of any universal model that would describe cell behavior well in all circumstances. For instance, a model that describes micropipette aspiration is not suitable for magnetic twisting cytometry (Wang et al. 1993). Even if one type of model fits two experiments, the material properties inserted into the model have to be very different (even by orders of magnitude) for each of the experimental cases. This discrepancy suggests that the models do not reflect what happens in the cell correctly. Smeets et al. (2019) suggest that one of the biggest flaws of all the above-described models is treating the cortex as a pseudo-two-dimensional body with no bending stiffness. They show a model which assumes that thick cortex and active tension can give good predictions of two processes: micropipette aspiration and two-cell aspiration. Similarly, Vargas-Pinto et al. (2013) discuss the discrepancy in the apparent moduli of a cell between experiments using spherical and sharp indenters. By finite element modeling, they show that the stiff cell cortex could explain this discrepancy.

### Mechanical properties of cell components

The mechanical properties of living cells have been studied for many years using various experimental methods. Micropipette aspiration (Mitchison and Swann 1954) is the oldest one. Later on, cytoindentation (Shin and Athanasiou 1999) and atomic force microscopy (Hoh and Schoenenberger 1994) gained popularity. The results of these experiments often yield significantly different results for individual cell components. The literature survey on cell component properties has been summarized in Supplementary Tables 1–4. We complement the information contained therein with two important observations.

First, the cytoplasm may be assumed liquid in very long time scales (> 10 s), but at shorter timescales the cytoplasm is viscoelastic (effective strain rates in the range of 0.1 s^−1^ < V/a < 2 s^−1^) and poroelastic at higher speeds (Hu et al. 2017). V/a is the ratio of velocity to the characteristic dimension. This ratio dictates the behavior of the material.

Second, the cortex properties are harder to obtain than other components. The cortex is around 200 nm thick and its Young’s modulus varies from 1 to 40 kPa (Maitre et al. 2012; Cartagena-Rivera et al. 2016).

### Hertz function and other models

The most common classic theory used to determine elasticity characteristics for indentation is by (Hertz, 1882). The aforementioned model was originally proposed to describe systems that satisfy some strong assumptions, including homogeneity of the material, isotropy, infinite thickness, small depth of indentation and deformations, specific probe geometry, etc.

The dependence of the force on the indentation in the Hertz model is given by:

1 $$F = \frac{4}{3}\frac{E}{{\left( {1 - \upsilon^{2} } \right)}}\sqrt r \delta^{3/2}$$

where *r* is the radius of the indenter, $E$ is Young’s modulus, δ is the indentation depth, and $\upsilon$ is the Poisson ratio.

The Hertz function is valid only for homogeneous materials. Extending the classic Hertz model to be applicable to indenting a layered spherical object—like a cell—is challenging. The case of a thick spherical shell was investigated by Berry et al. (2017). The authors provided a formula that allows for calculating a reaction force during the indentation of any spherical shell. However, this formula contains free parameters that have to be calculated using FEM.

The theories for flat-layered materials have already been developed (Stan and Adams 2016; Lee et al. 2018). However, an additional difficulty arises when the layered structure has nonzero Gaussian curvature, especially when the outer layer is stiffer than the inner layer. In that case, the geometrical-induced rigidity phenomenon (Lazarus et al. 2012) occurs. Its effects have been well studied. There is also a closed-form formula for describing a reaction force based on the mechanical properties of the consecutive layers. Unfortunately, the model is limited to the case of a relatively thin shell containing pressurized liquid (Vella et al. 2012). This model can be used to model plant cells (Beauzamy et al. 2015), which have rigid cell walls and are filled with cytoplasm. For animal cells, the cortex layer has stiffness comparable to the cytoplasm. Moreover, the cortex is relatively thicker. To the best of our knowledge, there is no generalization of multilayer theory for the mechanical conditions of an animal cell. In our approach, we check how well Hertz relation (1) described the simulated cell and we propose possible modifications of this relation to improve the compatibility.

## Materials and methods

Modeling was performed using the Finite Element Method (FEM) with the ANSYS Workbench/Mechanical environment. All simulations were performed using a static solver. Such an approach is valid for short time scales and small indentation depth when the cell behaves poroelastically (Hu et al., 2017) and its reaction is quasi-static (Nawaz et al. 2012).

The multiscale mesh was tetrahedral and fine-grained in the contact area between the cell and the spherical probe. In the radius of 0.7 µm around the contact point, the maximal mesh edge was 0.12 µm, and in the radius of 1.6 µm, no larger than 0.3 µm. These dimensions were chosen in such a way as to make the denser mesh comparable in size to the indenter radius**.** It was tested that further densification of the mesh does not change systematically the output of the simulation. The frictionless condition was chosen for the contact between the cell and the spherical probe.

**Fig. 1:**
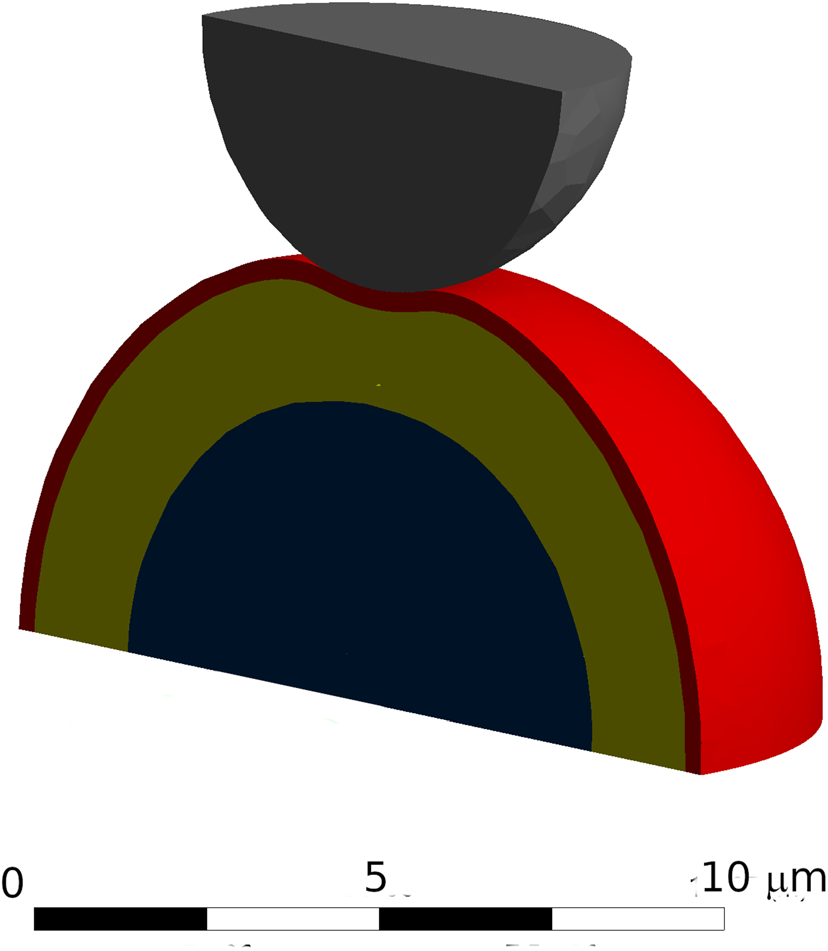
The general geometry for the simulation. The thin layer of cortex (red) surrounds the cytoplasm (yellow) and the immersed nucleus (blue). The spherical probe (grey) is inserted to a maximum depth of 0.5 µm

All modeled bodies were assumed to be an isotropic linear elastic material. This is an appropriate assumption for sub-micrometer indentation depth (Garcia 2020) and was chosen to avoid additional parameters associated with non-elastic behavior in the model. The cell was modeled as a hemisphere of the cytoplasm surrounded by the cortex (Fig. 1). The cell boundary condition for the flat bottom part was a fixed support. The indenter moved only in the vertical direction.

The cortex was treated as a three-dimensional solid to account for its bending stiffness. The physical parameters of most of the components (such as elasticity), as well as their sizes, varied in a credible range taken from the literature. The choice of range is justified in the previous section and in the supplementary tables. A set of average parameters was used as a reference for a typical setup (material properties were based on Bursa and Fuis 2010). The outer diameter of the hemisphere (cell) is equal to 13.2 µm. inside the cytoplasm there was a nucleus of **9.0** µm in diameter. The cytoplasm had a Young’s modulus of 0.25 kPa. The most external layer of the cortex, was 0.3 µm thick, and had a Young’s modulus of 5 kPa; the nucleus had a Young’s modulus of 1 kPa. The Poisson ratios of the cortex, cytoplasm, and nucleus were 0.3, 0.45, and 0.37, respectively. The indentation was performed by spherical indenters of various sizes, but the radius of 3.5 µm will be referred to as typical.

Such a model was chosen to minimize complexity while maintaining a realistic cell response to the AFM indentation. More complicated models were also tested. One of them included a 10 nm thick cell membrane. It turns out that the thin membrane does not contribute to the apparent elasticity of the cell. Therefore, in the final model, it was completely omitted. A similar observation was made for the nucleolus. Placing a 1.15 µm nucleolus (Young’s modulus of the order of kilopascals) inside the nucleus does not change the results noticeably. Changing Young’s modulus of the nucleus from 1 kPa to 0.45 kPa does not change the result either. Ultimately, the nucleolus was omitted and the Young’s modulus of the nucleus was kept constant at 1 kPa.

## Results and discussion

### General remarks

This section presents the results of the simulations performed using the model described in Sect. 3. The results were obtained in ca. 750 simulation runs for various combinations of parameters and an additional 750 for the case of a large nucleus described later. The variables characterizing the cell, like material properties and cortex thickness, varied over a realistic range. The elastic modulus of cortex and cytoplasm was changed from 1 to 10 kPa and 0.125 to 1 kPa, respectively. The thickness of the cortex was varied in the range of 0.1 to 0.8 µm and the radius of the indenter changed between 0.5 and 7 µm.

In the following sections, we investigate how the character of the cortex influences the measurements, we take under consideration different cell shapes and sizes, various nucleus sizes, different probe sizes, and we examine off-center load and friction. Finally, we propose a closed-form formula for the indentation force in the presence of the cortex and conduct a parametric study of its applicability.

### Initial validation

First, the results of our cell model were confronted with the simple Hertz model. As the Hertz model describes only homogeneous and isotropic materials, the cell model was simplified and the cell was made entirely of cytoplasm. The difference between the Hertz formula and the FEM model of such a cell was below 1% at every indentation depth. Therefore, we can assume that the Hertz model approximates the simple FEM model well. The modifications to the cell structure will be proposed in the following sections, and they will be confronted with the control Hertz model.

### Cortex as 3D body

**Fig. 2:**
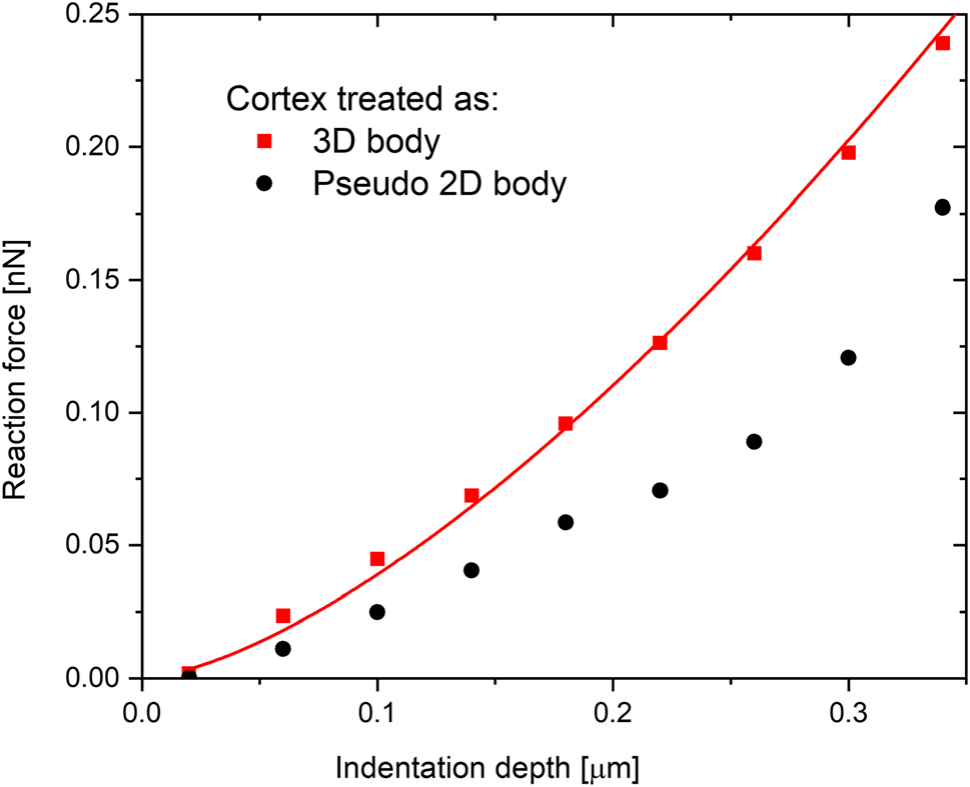
The dependence of the reaction force on the indentation depth. The line symbolizes the Hertz function fitted to the 3D body model

As mentioned previously in this paper, we treat the cortex as a solid 3D body. In this way, we can account for the influence of its bending stiffness in the results. To estimate the influence of bending stiffness on cell behavior, we compared the results obtained using two approaches to approximating the cortex. The first is the above-mentioned full 3D cortex with its real thickness; the second-modeling it as a thin 2-dimensional body with no bending stiffness. To mimic the 2D behavior, the cortex was actually modeled as an extremely thin 3D object so that the bending stiffness could be easily neglected. In this case, Young’s modulus was elevated to preserve the stretching stiffness of the cortex. The results are presented in Fig. 2. At first, the distinction may seem subtle, but at a small indentation depth, the reaction calculated using the full 3D model is twice as large as in the thin pseudo-2D model. This shows that the bending stiffness of the cortex cannot be neglected and therefore treating the cortex as a three-dimensional body is crucial.

Figure 2 presents an exemplary dependence of a reaction force on the indentation depth. The simulation was performed using our “typical” parameters, that is ${E}_{cor}$= 5 kPa and ${E}_{cyt}$= 0.25 Pa, cortex thickness of 0.3 µm, and the indenter radius of 3.5 µm. The fit from Fig. 2 shows good agreement with the Hertz function.

### Hertz-like model for a cell with cortex

A Hertz-like function was fitted to results obtained for various cortex thicknesses and indenter sizes to study how the cortex modifies the shape of the force-indent curve. By a Hertz-like function, we mean a function where the exponent *n* of the indentation (which normally is a constant value of 1.5) is treated as a free parameter:

2 $$F = A d^{n}$$

**Fig. 3:**
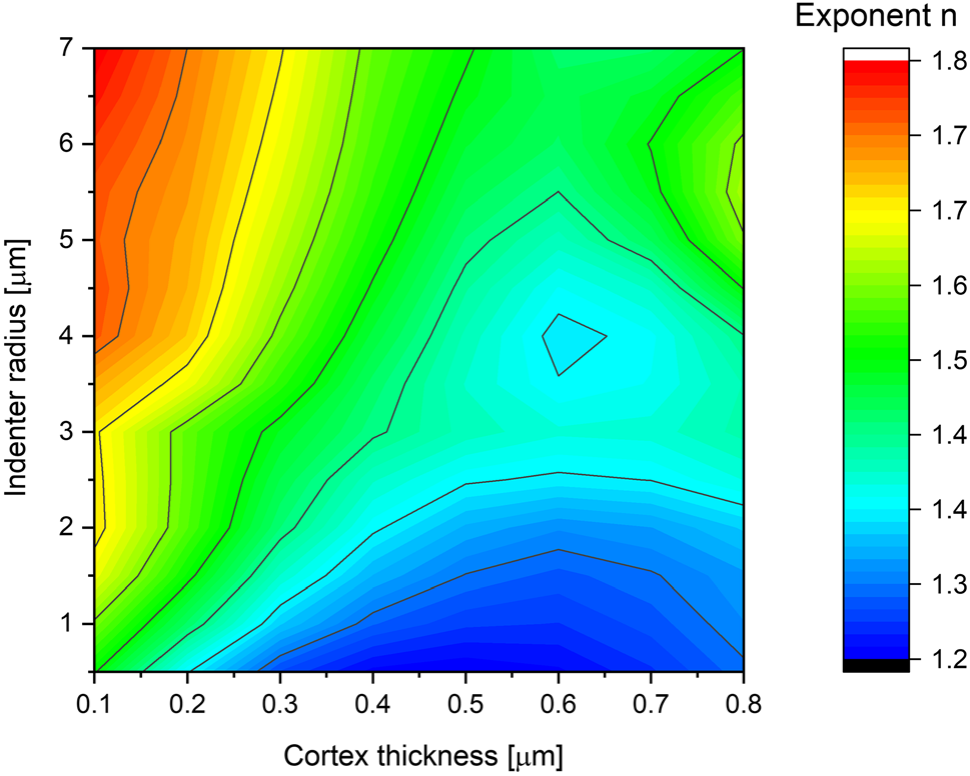
The dependence of the exponent ${\varvec{n}}$ from Hertz-like function on the cortex thickness and the indenter radius (Eq. 2)

In this series of fits, the constant $A$ was also treated as a free parameter *n* independently for all simulation parameter combinations. The dependence on the geometrical parameters for a typical cortex Young’s modulus is given in Fig. 3.

Finite element modeling shows that the standard exponent *n*=1.5 is the most appropriate for certain sets of cortex thicknesses and indenter radius. For all of the simulation runs exponent values ${\varvec{n}}$ ranged from 1 to almost 2, however, for the majority of simulations average values were close to 1.5. This result proves that in comparative studies of cells characterized by large variances of mechanical parameters, the Hertz function approximates the dependence on the indentation depth accurately.

### Cell size

**Fig. 4:**
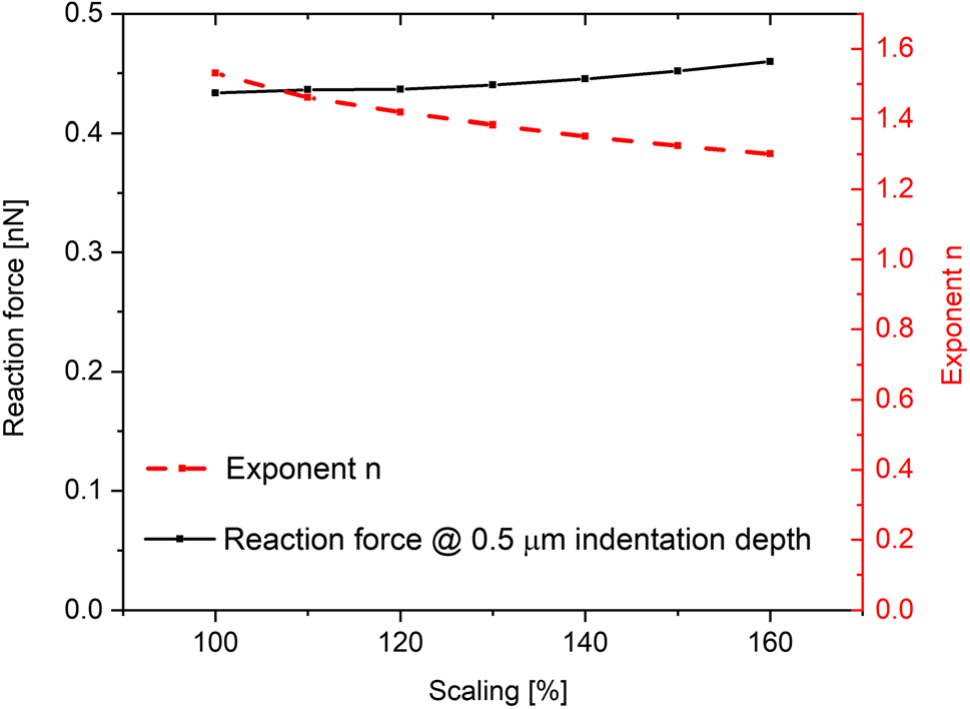
The dependence of the reaction force on the cell size

Another factor that was taken into consideration is the cell size. The results presented so far were obtained using our typical cell dimensions. Figure 4 presents the results obtained for a scaled-up cell. It appears that the influence of cell size on the results is not very pronounced. Moreover, in our simulations cell size is the only geometrical parameter that is not scanned over. This means it can be omitted and the results can be obtained from scaling existing results. One can think of the ratio of cell size to indenter size as the vital parameter. The results for the scaled-up cell are equivalent to the results with a smaller indenter multiplied by the scale factor squared.

That way we can assume that formula (4) encapsulates all geometrical factors important for the indentation experiment on the cell with small to moderate nucleus size. The case of the large nucleus is treated separately in Sect. 4.8. The free parameters in the formula are a solely function of the material properties. The use of such a formula could be very useful when one wants to measure changes in the cell material properties due to some factor, because it allows one to omit the influence of the cell’s geometry. The relevance of other geometrical parameters is tested in the following sections.

### Cell shape

**Fig. 5:**
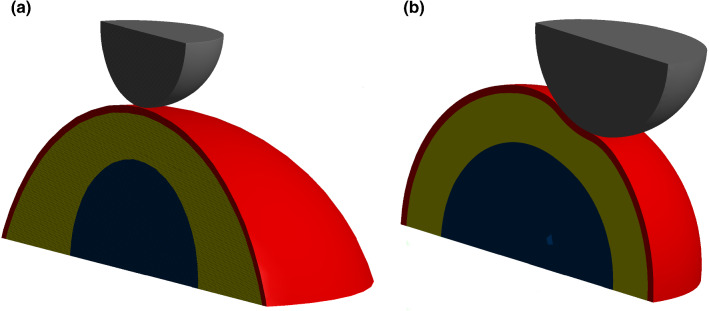
**a** Central indentation of a model cell with varying cytoplasm thickness. **b** Non-central indentation of the spherical cell

**Fig. 6:**
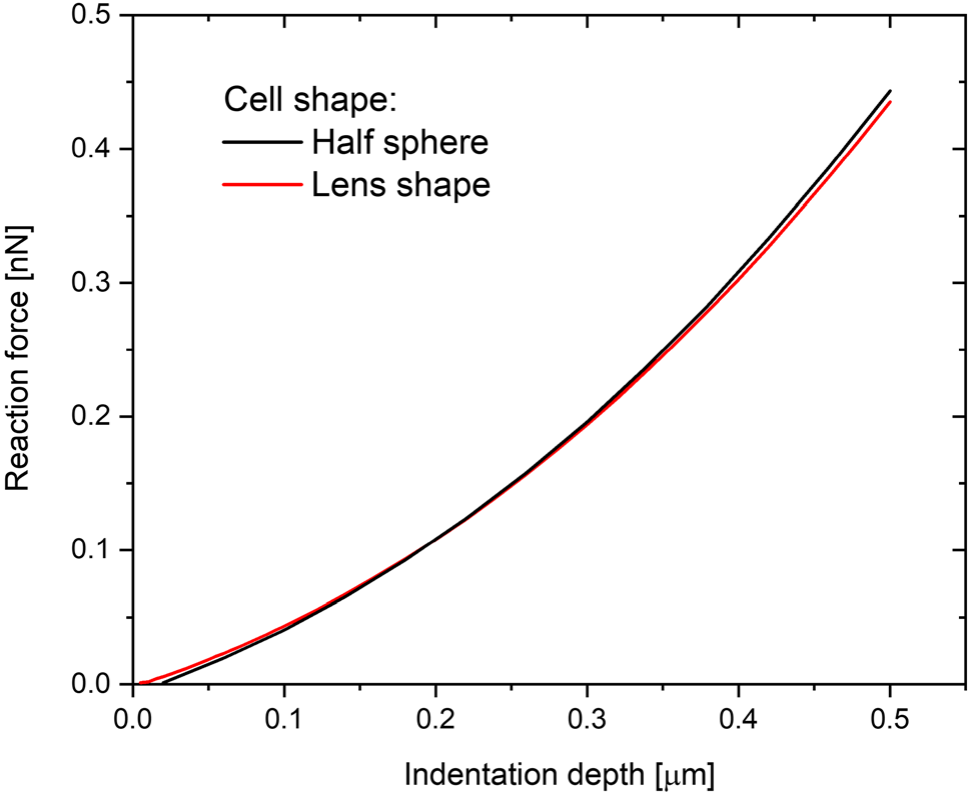
Comparison of the indentation of our typical hemispherical cell (Fig. 1) and the flattened one (Fig. 5a)

Apart from the cell size, we also investigate the cell shape. The thickness of the cytoplasm varies in different parts of the cell, so the nucleus and cortex are not concentric spheres. To estimate the influence of the cytoplasm layer and the curvature of the cell cortex, two simulations were compared. One was our typical cell, and the other was a cell with the same height only with a 50% larger radius of curvature that maintained the same overall height of the cell. The cortex thickness and nucleus were the same for both cases (Fig. 5a). The simulation shows that the lens-like cell with a larger radius of curvature varied marginally from the typical dome cell (Fig. 6).

### Off–center load and friction

**Fig. 7:**
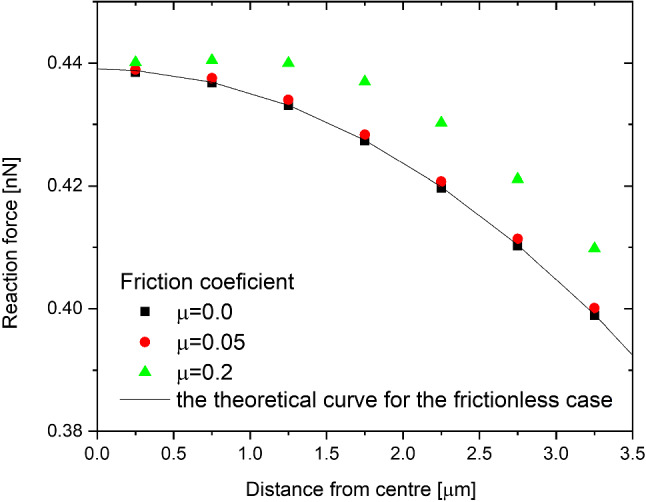
The effects of friction and non-central indentation on the maximal reaction force for constant vertical indentation depth (in the *y* direction). The black squares correspond to the situation without friction, the red squares correspond to realistic friction (based on experiments), and the green squares correspond to the largely overestimated friction coefficient. On the *x-axis,* there is a horizontal distance between the center of the cell and the center of the indenter. The solid line represents the theoretical model without friction using Eq. (3)

The effects of friction and non-central indentation are discussed in this section. To investigate this effect, a set of simulations was performed. The coefficient of friction and horizontal distance between the center of the cell and the center of the indenter were varied (Fig. 5b). The friction coefficient turns out to be important only if the cell is not indented axially. What is more, in reality the coefficient is very small, ranging from µ=0.03 to µ=0.06 (Dunn et al. 2007; Angelini et al. 2012). In this range, friction has a minimal effect even for non-axial indentation (Fig. 7). Without the presence of friction, the effect of non-central indentation force can be easily calculated based on the assumption that only radial force—normal to the surface—is present. The shear stress vanishes. In this case, the only factor that dictates the reaction force is the radial indentation depth. The indentation curve calculated in the center of the cell can be calculated according to the following formula:

3 $$i^{\prime } = R + r - \sqrt {a^{2} + \left( {\sqrt {\left( {R + r} \right)^{2} - a^{2} } - i} \right)}$$

where *i ‘* is the effective indentation, *i*—indentation in the *y* direction (vertically), *R*, *r*—cell and indenter radii, respectively, and *a*—horizontal distance between the center of the cell and the center of the indenter (offset). The formula is derived in the appendix.

### Dependence on nucleus size

**Fig. 8:**
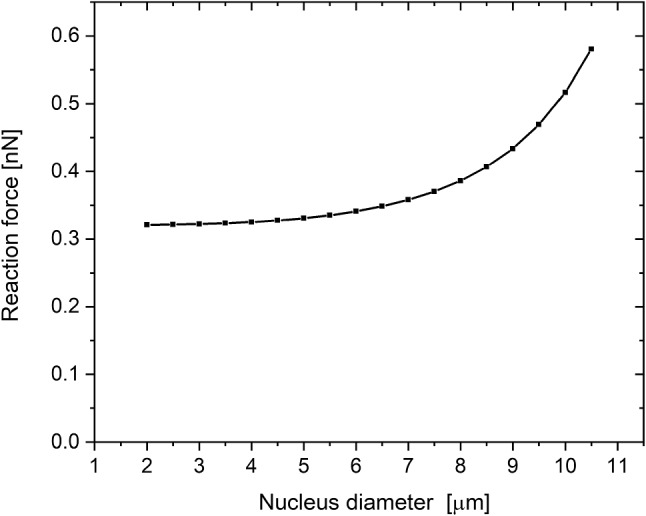
The reaction force for 0.5 µm indentation depth for typical parameters as a function of the nucleus diameter

The nucleus size is a factor that varies between different cell types. Therefore, to check the influence of that parameter on the indentation, additional series of simulations were performed. In the simulations, the nucleus diameter was swept over the range from 10,5 µm to 2 µm. It appears that the nucleus size has a significant influence on the reaction force for a large nucleus. For diameters smaller than 9 μm, the dependence flattens (Fig. 8). This dependence is easy to understand. With an indentation depth of 0.5 µm, it is impossible to probe a nucleus whose surface is 2 μm below the surface of the cell or deeper. All the previous analyses were performed for a nucleus radius of 4.5 µm.

As there is little dependence on nucleus size smaller than 4.5 µm, this analysis is valid for any smaller nucleus size.

Calculations for the special case of the big nucleus were also performed. The chosen size was 10.5 µm. The result is presented in Sect. 4.11.

### Dependencies on the geometrical quantities

**Fig. 9:**
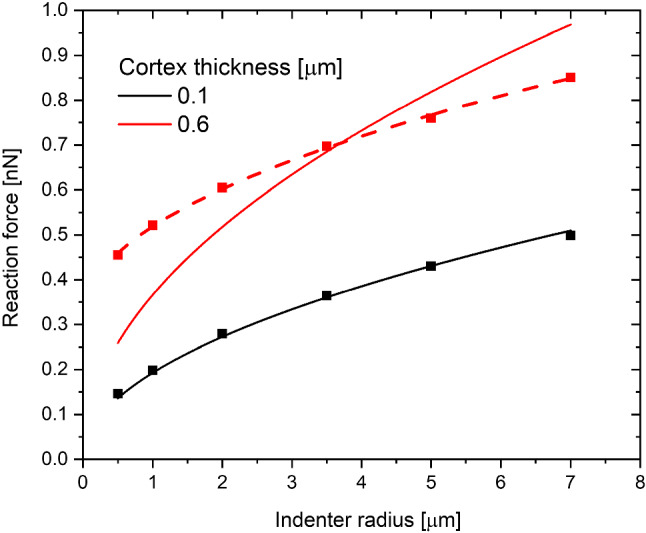
Dependence of the reaction force at 0.5 µm on the indenter radius. The solid lines represent the Hertz function fit, and the dashed line is the Hertz function shifted vertically

In Sect. 4.4, it was shown that the dependence on the indentation depth can be approximated as a constant (which we call $A$) multiplied by a power function. The exponent of the function is close to the value of 1.5, as in the Hertz mode, and does not depend sharply on the cell properties or the experimental conditions. Thus, these dependencies must be contained in the constant $A$. In our study, the power functions Eq. (2) with an exponent of 1.5 were fitted to the simulation results. The resulting apparent moduli are shown in Fig. 9

**Fig. 10:**
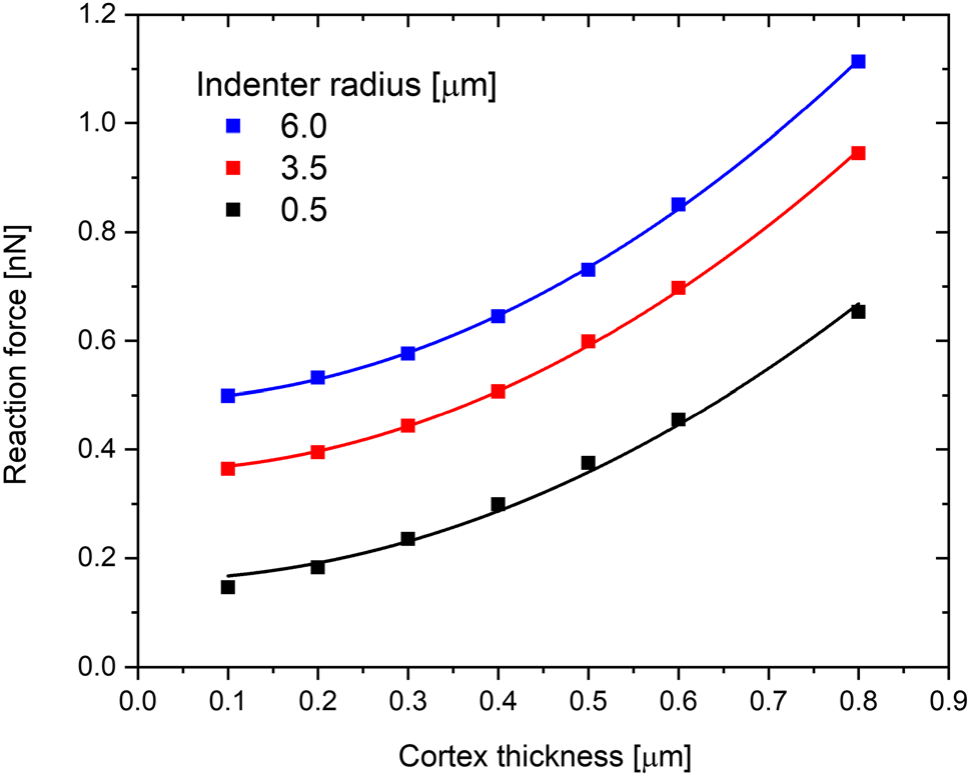
The dependence of the reaction force at 0.5 μm depth on the thickness of the cortex. Solid lines represent the fit of the square function to the data (${{\varvec{y}}}_{0}+{{\varvec{y}}}_{1}\boldsymbol{*}{{\varvec{t}}}^{2}-$where y_0_ and y_1_ are fit parameters)

The original Hertz function has a square-root dependence on the indenter radius. As can be seen in Fig. 9, the Hertz function works well only for a very thin cortex. With an increase in the cortex thickness, the function fails to describe the dependence on the indenter radius. The dependence for the thick cortex looks like a square-root dependence of the Hertz function shifted by a certain value. In order to examine this shift closely, the dependence of reaction force on the cortex thickness was plotted (Fig. 10). As can be seen from this plot, the shift can be well described by the square function of the cortex thickness.

All of the above-mentioned observations allow us to write down the dependence of the reaction force on all geometrical factors. We have shown in the previous section that the dependence on the indentation depth $d$ could be approximated as ${d}^{3/2}$. Combining this square dependence on the cortex thickness with the square-root dependence on the indenter radius from Hertz function yields the dependence of the reaction force on the geometrical properties of the system,

4 $$F = E^{*} \left( {r^{\frac{1}{2}} + p*t^{2} } \right)d^{3/2}$$

Here $t$ is cortex thickness and $p$ is a free parameter. In fits shown in Fig. 3, we treat ${E}^{*}$ as a free parameter for the pure Hertz function fitting. The function Eq. (4) has a simple form, only slightly more complicated than the original Hertz function. Yet for a vast parameter range, it offers a large improvement in the accuracy. The function very well approximates the combined dependence of the reaction force on the cortex thickness and the radius of the indenter. The next section presents a study that checks how well this function approximates the experimental results.

#### Parametric study

In this subsection, we determine the range of cell properties in which our modified Hertz function is accurate. The proposed function Eq. (4) was fitted to all simulation results. The free parameters in this function ( $${E}^{*}$$ and $$p)$$ do not depend on the geometrical factors, they were kept constant for the given cortex and cytoplasm Young’s moduli combination. These parameters depend solely on the properties of the material.

Assessment of the value of fit quality using R-squared allows one to determine the parameter range for which the proposed parametrizing function is valid. The function generally fits quite well, especially for the lower half of cytoplasm Young’s moduli range. The direct comparison of the global resulting formulas (4 and 5) to the data is given in Sect. 4.12.

**Fig. 11:**
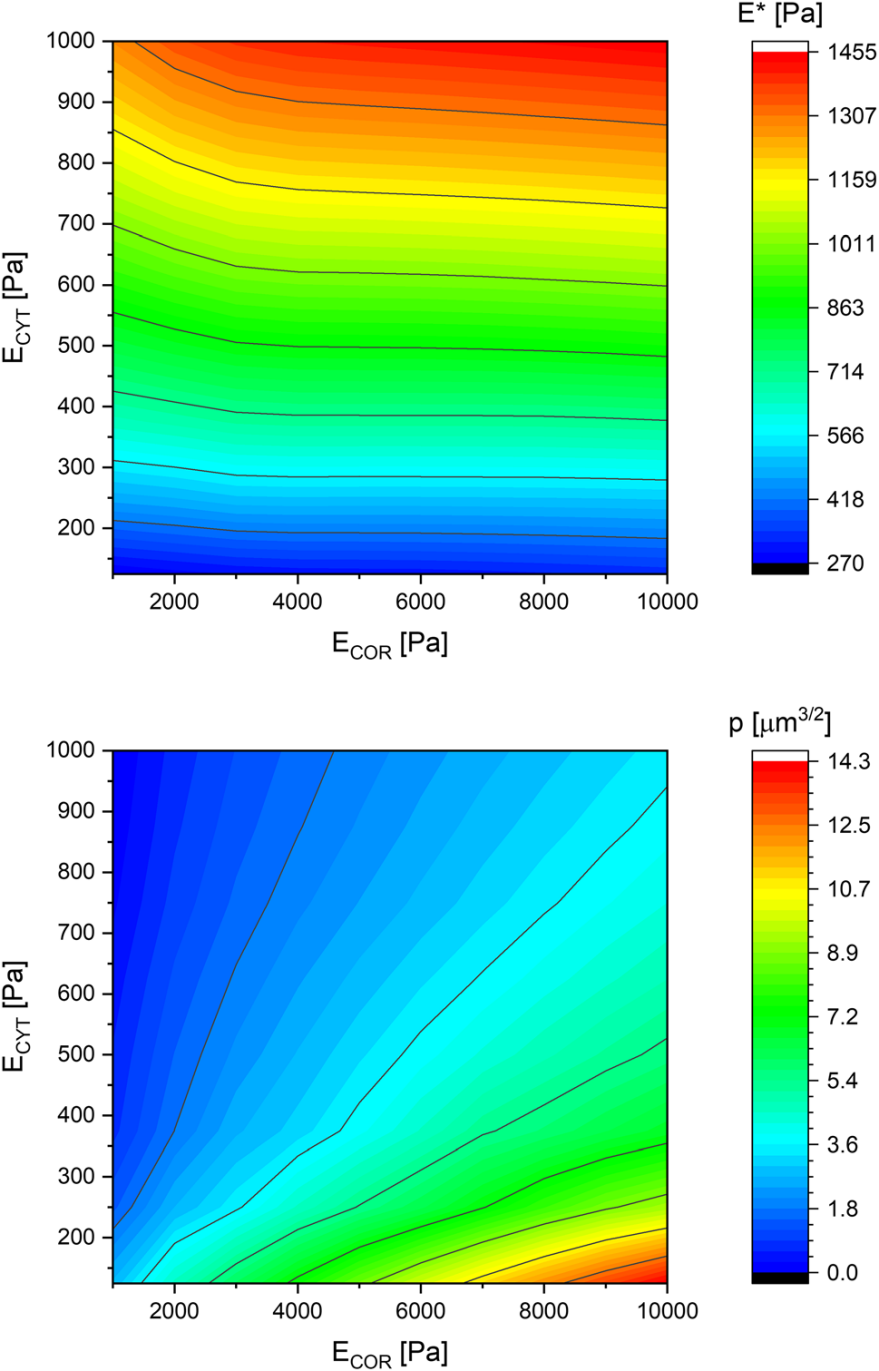
Dependence of the modified Hertz function parameters on Young’s moduli

Figure 11 shows the dependence of fitted parameters on the simulated data. Generally, the dependencies are slower than linear. Some effort was put into the functionalization of these dependencies in a simple form. It was found that the parameters ${E}^{*}$ and $p$ can be expressed as Eq. (5). After being inserted into Eq. (4), these relations approximate the dependence very well.

5 $$\begin{aligned}E^{*} = 7\left[ {Pa} \right]^{1/4} E_{CYT}^{3/4} + 0.069\sqrt {E_{CYT} E_{COR} } \\ p = 0.66\left[ {\mu m} \right]^{ - 3/2} \left( {\frac{E_{COR} }{E_{CYT} }} \right)^{{0.704}} ,\end{aligned}$$

where ${E}_{cyt}$ and ${E}_{cor}$ are Young’s moduli of cytoplasm and cortex.

#### Large nucleus

As mentioned earlier, a similar analysis was conducted for the case of a large nucleus. Another ca. 750 simulations runs were performed, scanning the same range of parameters as previously. The difference was that the diameter of the nucleus was now considerably larger (10.5 μm). The results were analyzed in the same manner as described in Sects. 4.6 and 4.8. They showed overall trends similar to those obtained with a smaller nucleus. Formula (4) fits the data exceptionally well when ${{\varvec{E}}}_{{\varvec{C}}{\varvec{O}}{\varvec{R}}}$ is 2 to 8 kPa and ${{\varvec{E}}}_{{\varvec{C}}{\varvec{Y}}{\varvec{T}}}$ is 0.2 to 0.5 kPa. For the rest of the data, the fit is also as satisfactory as for the standard nucleus size. However, as shown in Fig. 8, the larger nucleus makes the cell considerably stiffer, so the formulas for parameters Eqs. (5) will not accurately describe the behavior of a large cell. Once again, the parameters of formula (4) were optimized for the large cell case, yielding:

6 $$\begin{aligned} E^* &= 37.7*\left[ {Pa} \right]^{{\frac{1}{2}}} E_{{CYT}}^{{\frac{1}{2}}} + 0.102\sqrt {E_{{CYT}} E_{{COR}} } \\ &{\,\,}{}p = 0.59\left[ {\mu m} \right]^{{ - 3/2}} \left( {\frac{{E_{{COR}} }}{{E_{{CYT}} }}} \right)^{{0.667}}\end{aligned}$$

#### Quality of the approximation

**Fig. 12:**
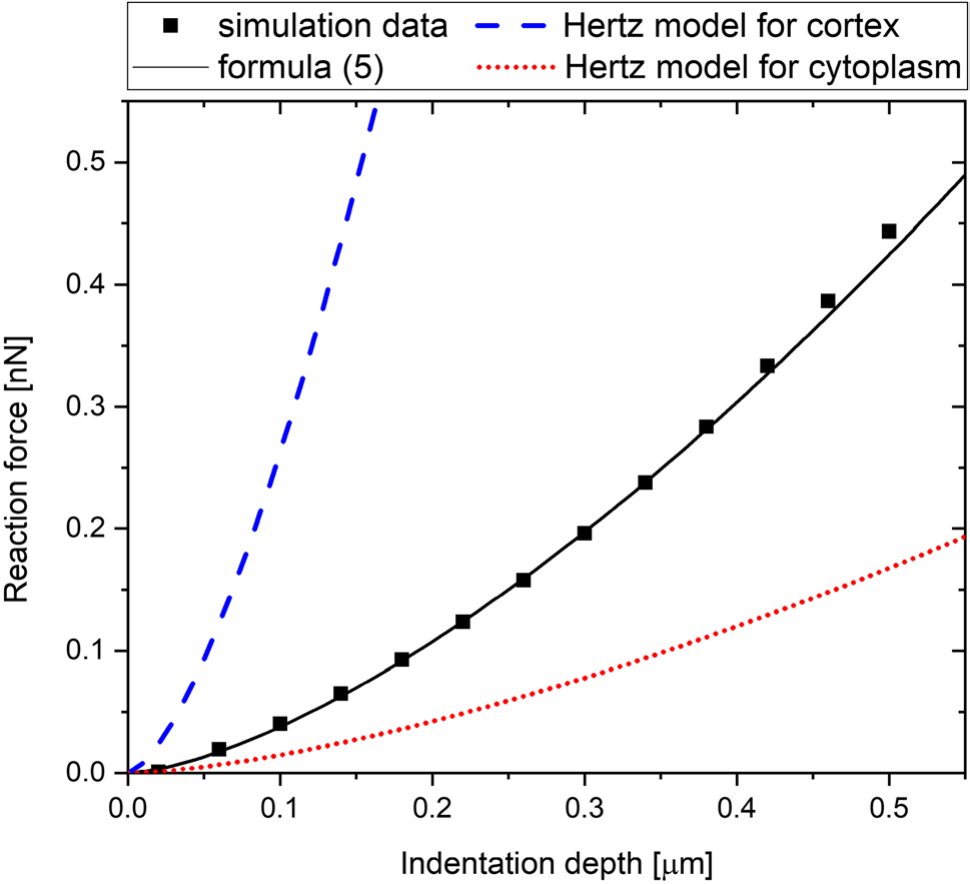
Comparison of the formula (4) with (5) approximations to the Hertz models for *E*_CYT_=250 Pa, *E*_COR_=5000 Pa, *R* =3.5 µm, *t* = 0.3 µm

**Fig. 13:**
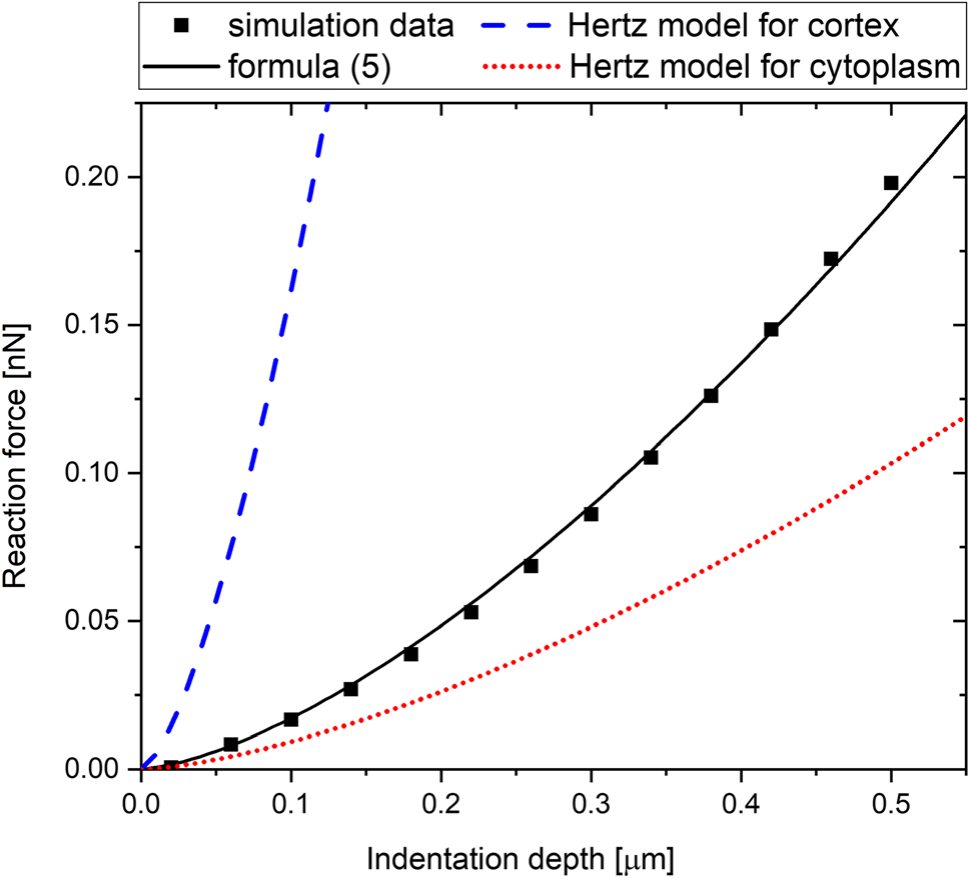
Comparison of the formula (4) with (5) approximations to the Hertz models for *E*_CYT_ = 250 Pa, *E*_COR_=5000 Pa, *R* =1 µm, *t* = 0.1 µm

**Fig. 14:**
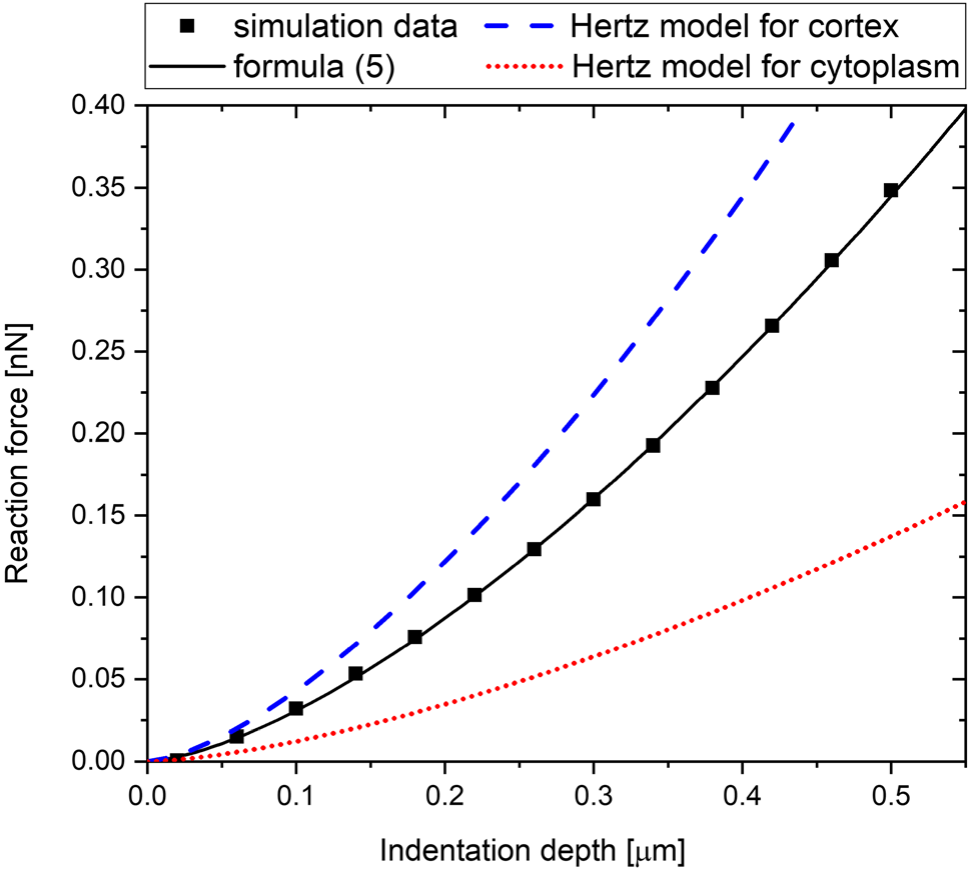
Comparison of the formula (4) with (5) approximations to the Hertz models for *E*_CYT_ = 250 Pa *E*_COR_ = 1000 Pa, *R* =2 µm, *t* = 0.6 µm

**Fig. 15:**
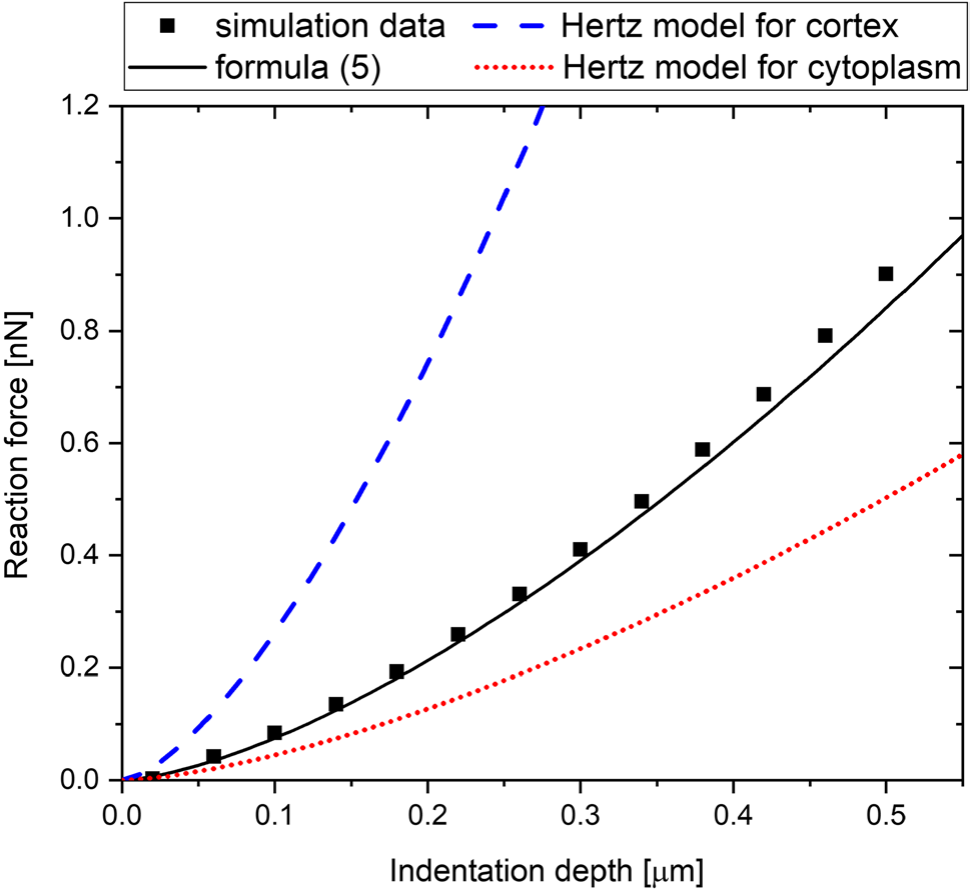
Comparison of the formula (4) with (5) approximations to the Hertz models for *E*_CYT_ = 750 Pa, *E*_COR_ = 5000 Pa, *R* =3.5 µm, *t* = 0.3 µm

In this section, the accuracy of the proposed formula is tested. Figures 12, 13, 14 and 15 show how well relations (5) substituted into the formula (4) reflect the results, compared to the Hertz model. The Hertz model works for uniform materials, so for comparison we added the Hertz model for the cytoplasm and the cortex. The examples were selected to cover most of the parameter range. For the typical values of the parameters (Fig. 12) formula (4) fits the data adequately, while the Hertz function with the Young’s modulus of the cortex is too stiff, and with the Young’s modulus of the cytoplasm it is too soft. The same situation occurs when we take a different point from the simulation parameter space with a stiffer cytoplasm (Fig. 15). However, for the point with a very thin cortex (Fig. 13), our formula is not as precise, but still much better than the Hertz function with the Young’s modulus of the cytoplasm. Let us look at the case of another extreme. For a thick cortex with a fairly low Young’s modulus (Fig. 14), we find that the formula still approximates the simulation well, but the Hertz model with Young’s modulus of the cortex is only slightly off. This can be explained by the fact that the cytoplasm cannot be probed through such a thick cortex.

Previously, no closed-form formula was proposed to include the properties of the cortex in an animal cell indentation experiment. The proposed formula (4) has some characteristic of a model describing an elastic ball and a spherical shell. The first part of the proposed formula is similar to the Hertzian contact. They have the same square root dependence on the indenter radius. The second part of the formula is reminiscent of the shell theory. The square dependence on the shell thickness is the same as in classical Reissner theory (Reissner 1946). Although for unpressurized shells the dependence gets slightly sharper for deeper indentations (Pogorelov 1988). When we consider the pressure in the cells, the Reissner theory can be extended to the deeper indentations just by introducing an effective Young’s modulus (Lazarus et al. 2012). Therefore, the dependence holds even for deep indentation for a lightly pressurized shell. The cytoplasm will exert some pressure on the cortex, so formula (4) can be interpreted as the sum of the Hertzian contact for the ball and the Lazarus dependence for the lightly pressurized shell indentation.

The proposed formula does not contain any free parameters, thus it can be used to extract material parameters (Young’s moduli of cytoplasm and cortex, more specifically the cube root of cortex Young’s modulus multiplied by its thickness). This does not allow us to fully characterize all mechanical properties of the cell, but gives a much better insight into the cell structure than using the Hertz formula. The Hertz formula yields only apparent Young’s modulus, which is not directly associated with any of the fundamental properties of the cell material or structure.

## Conclusions

This work focuses on modeling the indentation of the animal cell, while treating the cortex as a full three-dimensional body with its real bending stiffness. It shows that the dependence of the reaction force on the indenter radius is similar to the one from the Hertzian contact, only shifted vertically by a term proportional to the squared thickness of the cortex. In other words, the dependence of the reaction force on the cortex thickness is a square function. One can imagine that during indentation, when the cortex ‘surrounds the probe’, it changes the size of the probe. By adding an extra term, we managed to regain Hertz-like behavior with much better convergence of the model to the experimental data, and we maintained the simplicity of the proposed equations. It is also important to emphasize that the proposed model modification is not based on simply increasing complexity by adding a few free parameters, but on introducing corrections related to material data.

Moreover, there is a significant difference in response when treating the cortex as 3D in contrast to the 2D body without bending stiffness. The difference is more pronounced for smaller indentation depths. For our typical cell, there was a twofold relative difference in the applied force at a small indentation depth.

On the other hand, the influence of friction and off-center load is less significant. It turns out that the friction of the order of magnitude measured in the experiment does not contribute much to the force-indentation curve. Similarly, the maximal force measured under constant vertical indentation depth changes little between the center and the side of the cell. We presented the formula for computing the effective indentation depth, knowing the distance from the center and the vertical indentation.

## Supplementary Information

Below is the link to the electronic supplementary material.

Supplementary file1 (DOCX 93 kb)
