# Supplementary material for: The role of the cortex in indentation experiments of animal cells
Source: Biomech Model Mechanobiol. 2022 Oct 25;22(1):177–87. doi: 10.1007/s10237-022-01639-5 (PMC9958175; doi:10.1007/s10237-022-01639-5)
Supplement: Supplementary file 1 — Supplementary file1 (DOCX 93 kb) [file 10237_2022_1639_MOESM1_ESM.docx]

Appendix to :The Role of the Cortex in Indentation Experiments of Animal Cells

Leszek Krzemien, Magdalena Giergiel, Agnieszka Kurek, Jakub Barbasz

# Derivation of formula (3)

.
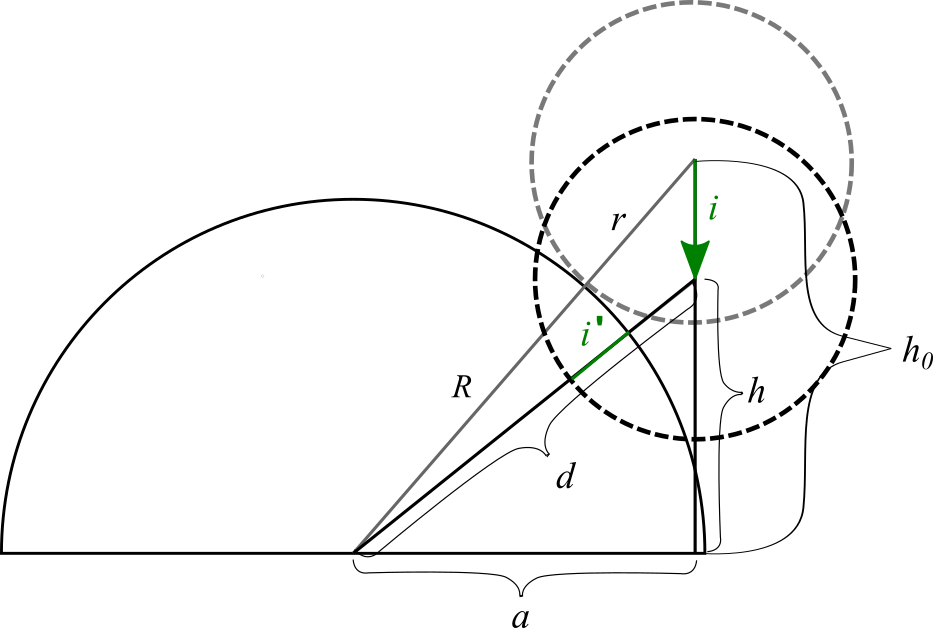


Figure 1. Geometry of the eccentric indentation

i’ it the real indentation depth that we are looking for.

From fig.1 we see that:

$h=h_{o}-i$, (1)

from the Pythagoras equation we obtain

$h_{0}= \sqrt{\left( R+r \right)^{2}-a^{2}}$. (2)

$d$ can be expresses as

$d=\sqrt{a^{2}+h^{2}}$, (3)

but also as

$d=R+r-i',$ (4)

so:

$i'=R+r-d$. (5)

Substituting (1),(2) and (3) into (5) we obtain:

$i'=R+r-\sqrt{a^{2}+\left( \sqrt{\left( R+r \right)^{2}-a^{2}}-i \right)}$. (6)

# Tables

### Table 1. Membrane properties

| Publication | Technique | Cell Type | Young’s Modulus | Poisson ratio |
| --- | --- | --- | --- | --- |
| Bursa and Fuis (2010) | AFM | Smooth muscle cells | 10 kPa | 0.3 |
| McGarry and Prendergast (2004) | Loading using horizontal and vertical forces | Adherent fibroblasts | 1 kPa | 0.3 |
| Hochmuth, Mohandas et al. (1973) | Fluid mechanical technique | Red cells | 1 kPa  (10 nm thickness) | - |

### Table 2. Cortex properties

| Publication | Technique | Cell type | Elasticity | Poisson ratio |
| --- | --- | --- | --- | --- |
| Bursa and Fuis (2010) | AFM | Smooth muscle cells | 5 kPa | 0.3 |
| Cartagena-Rivera, Logue et al. (2016) | AFM | Nonadherent  fibroblast | 22 +/- 5 kPa  42+/-9 kPa | - |
| Rotsch and Radmacher (2000) | AFM | Adherent fibroblasts | 10-100 kPa | - |
| Bausch, Ziemann et al. (1998) | Magnetic bead microrheometer | Adherent fibroblasts | Shear modulus  20-40 kPa | - |
| Nawaz, Sánchez et al. (2012) | Optical trap, AFM | Mouse embryonic fibroblasts | 100.3 +/- 10.2 Pa opt. trap  85.3+/-4.5 Pa AFM  High force: 140 Pa slow; 330 Pa fast | 0.4 |

### Table 3. Cytoplasm properties

| Publication | Technique | Cell Type | Elasticity | Poisson ratio |
| --- | --- | --- | --- | --- |
| Liu et al., (2019) | AFM | Mouse embryonic fibroblasts | Shear modulus  G_0_ = 145 Pa  E=83,77+/- 14 Pa | 0.37 |
| Bursa and Fuis (2010) | AFM | Smooth muscle cells | 250 Pa | 0.45 |
| McGarry and Prendergast (2004) | Loading using horizontal and vertical forces | Adherent fibroblasts | 100 Pa | 0.37 |
| Caille, Thoumine et al. (2002) | Compression between microplates | Endothelial cells | ~500 Pa  323 Pa round cell  775 Pa spread cell | 0.5 |
| Shin and Athanasiou (1999) | Cytoindentation | MG63 osteoblast-like cells | Aggregate modulus of 2.05 +/- 0.89 kPa  Shear modulus  0.41 +/- 0.17 | 0.37 +/- 0.03 |
| Trickey, Lee et al. (2000) | Micropipette aspiration | Chondrocytes | Equilibrium modulus k1=240 +/-11 Pa  Instantaneous modulus k1+k2=410+/-17 Pa  Young’s modulus E=360 Pa | 0.4 |

### Table 4. Nucleus properties

| Publication | Technique | Cell type | Young’s Modulus | Poisson ratio |
| --- | --- | --- | --- | --- |
| Liu, Mollaeian et al. (2019) | AFM | Mouse embryonic fibroblasts | 395+/-50 Pa | 0.37 |
| Bursa and Fuis (2010) | AFM | Smooth muscle cells | 1000 Pa | 0.3 |
| McGarry and Prendergast (2004) | Loading using horizontal and vertical forces | Adherent fibroblasts | 400 Pa | 0.37 |
| Caille, Thoumine et al. (2002) | Compression between microplates | Endothelial cells | ~5000 Pa  Isolated 8000 Pa | 0.5 |
| Guilak, Tedrow et al. (2000) | Micropipette aspiration | Articular chondrocytes | 1000 Pa | 0.5 |

Bausch, A. R., F. Ziemann, A. A. Boulbitch, K. Jacobson and E. Sackmann (1998). "Local measurements of viscoelastic parameters of adherent cell surfaces by magnetic bead microrheometry." Biophys J **75**(4): 2038-2049.

Bursa, J. and V. Fuis (2010). Finite element simulation of mechanical tests of individual cells. **25:** 16-19.

Caille, N., O. Thoumine, Y. Tardy and J. J. Meister (2002). "Contribution of the nucleus to the mechanical properties of endothelial cells." J Biomech **35**(2): 177-187.

Cartagena-Rivera, A. X., J. S. Logue, C. M. Waterman and R. S. Chadwick (2016). "Actomyosin Cortical Mechanical Properties in Nonadherent Cells Determined by Atomic Force Microscopy." Biophys J **110**(11): 2528-2539.

Guilak, F., J. R. Tedrow and R. Burgkart (2000). "Viscoelastic Properties of the Cell Nucleus." Biochemical and Biophysical Research Communications **269**(3): 781-786.

Hochmuth, R. M., N. Mohandas and P. L. Blackshear, Jr. (1973). "Measurement of the elastic modulus for red cell membrane using a fluid mechanical technique." Biophysical journal **13**(8): 747-762.

Liu, Y., K. Mollaeian and J. Ren (2019). "Finite element modeling of living cells for AFM indentation-based biomechanical characterization." Micron **116**: 108-115.

McGarry, J. G. and P. J. Prendergast (2004). "A three-dimensional finite element model of an adherent eukaryotic cell." Eur Cell Mater **7**: 27-33; discussion 33-24.

Nawaz, S., P. Sánchez, K. Bodensiek, S. Li, M. Simons and I. A. T. Schaap (2012). "Cell Visco-Elasticity Measured with AFM and Optical Trapping at Sub-Micrometer Deformations." PLOS ONE **7**(9): e45297.

Rotsch, C. and M. Radmacher (2000). "Drug-induced changes of cytoskeletal structure and mechanics in fibroblasts: an atomic force microscopy study." Biophys J **78**(1): 520-535.

Shin, D. and K. Athanasiou (1999). "Cytoindentation for obtaining cell biomechanical properties." J Orthop Res **17**(6): 880-890.

Trickey, W. R., G. M. Lee and F. Guilak (2000). "Viscoelastic properties of chondrocytes from normal and osteoarthritic human cartilage." Journal of Orthopaedic Research **18**(6): 891-898.
